# Hepatic Xenobiotic Metabolizing Enzyme and Transporter Gene Expression through the Life Stages of the Mouse

**DOI:** 10.1371/journal.pone.0024381

**Published:** 2011-09-08

**Authors:** Janice S. Lee, William O. Ward, Jie Liu, Hongzu Ren, Beena Vallanat, Don Delker, J. Christopher Corton

**Affiliations:** 1 National Health and Environmental Effects Research Laboratory, United States Environmental Protection Agency, Research Triangle Park, North Carolina, United States of America; 2 University of Kansas Medical Center, Kansas City, Kansas, United States of America; Hokkaido University, Japan

## Abstract

**Background:**

Differences in responses to environmental chemicals and drugs between life stages are likely due in part to differences in the expression of xenobiotic metabolizing enzymes and transporters (XMETs). No comprehensive analysis of the mRNA expression of XMETs has been carried out through life stages in any species.

**Results:**

Using full-genome arrays, the mRNA expression of all XMETs and their regulatory proteins was examined during fetal (gestation day (GD) 19), neonatal (postnatal day (PND) 7), prepubescent (PND32), middle age (12 months), and old age (18 and 24 months) in the C57BL/6J (C57) mouse liver and compared to adults. Fetal and neonatal life stages exhibited dramatic differences in XMET mRNA expression compared to the relatively minor effects of old age. The total number of XMET probe sets that differed from adults was 636, 500, 84, 5, 43, and 102 for GD19, PND7, PND32, 12 months, 18 months and 24 months, respectively. At all life stages except PND32, under-expressed genes outnumbered over-expressed genes. The altered XMETs included those in all of the major metabolic and transport phases including introduction of reactive or polar groups (Phase I), conjugation (Phase II) and excretion (Phase III). In the fetus and neonate, parallel increases in expression were noted in the dioxin receptor, Nrf2 components and their regulated genes while nuclear receptors and regulated genes were generally down-regulated. Suppression of male-specific XMETs was observed at early (GD19, PND7) and to a lesser extent, later life stages (18 and 24 months). A number of female-specific XMETs exhibited a spike in expression centered at PND7.

**Conclusions:**

The analysis revealed dramatic differences in the expression of the XMETs, especially in the fetus and neonate that are partially dependent on gender-dependent factors. XMET expression can be used to predict life stage-specific responses to environmental chemicals and drugs.

## Introduction

Detoxification and elimination of drugs, environmentally-relevant chemicals and endogenous metabolites is a major function of the liver and important in maintaining the metabolic homeostasis of the organism. Xenobiotics are metabolized by a large number of xenobiotic metabolizing enzymes (XMETs) which fall into three broad categories: phase I, phase II and phase III. Phase I enzymes are mainly monooxygenases that convert hydrophobic xenobiotics into hydrophilic molecules and include cytochrome P450 family members, alcohol and aldehyde dehydrogenases, and amine oxidases. The cytochrome P450 (CYP) enzymes catalyze oxidative metabolism of a vast number of compounds, including many proteratogens, procarcinogens, and promutagens to reactive and toxic intermediates. Phase II enzymes convert the products of phase I metabolism into amphiphilic anionic conjugates that are water soluble and include glutathione transferases, UDP-glucuronyl transferases, and sulfotransferases. Phase III genes export conjugated xenobiotics out of the liver and include ATP binding cassette subfamily members, organic anion and cation transporters, and solute carriers [1]. A large number of genetic and biochemical studies have shown that the level of expression and activity of individual XMETs in part, determines the fate of a specific xenobiotic and whether exposure results in toxicity [2] [3].

Pharmacokinetic differences between the fetus, newborns, children, and the aged may alter responses to chemicals compared to adults, potentially resulting in differences in therapeutic drug efficacy and for environmentally-relevant chemicals, adverse health effects. Numerous changes take place in the liver during the fetal and neonatal period. Hematopoiesis, which is a major function of the fetal liver, declines dramatically during liver maturation as hematopoietic stem cells migrate elsewhere [4]. During the late fetal and neonatal stages, the liver initiates the expression of genes associated with liver maturation and starts forming the architecture of the liver lobules. Associated with these changes, hepatocytes start expressing various types of XMETs including *CYP* genes [5] [6] [7]. Many environmental chemicals and drugs are known to cause unwanted effects in the embryo or fetus, including in utero death and birth defects which are determined in part by XMET expression. Likewise, in the elderly, there are pharmacokinetic changes that are attributed in part to decreases in the volume of the liver and diminished hepatobiliary functions including decreases in phase I drug metabolism capability [8]. Dramatic changes in the expression of some XMETs have been observed in the livers of aged rats [9] [10]. The prediction of responses to chemicals and drugs in the elderly is complicated by the fact that the elderly population has a burden of or is more susceptible to various diseases and are often prescribed several drugs concurrently.

No systematic analysis of the expression of XMETs through different life stages has been carried out in any species to determine differences with adults. Knowledge of XMET mRNA expression would be a useful starting point to predict chemical metabolism and associated responses as a function of life stage. Here, we used full-genome microarrays to comprehensively identify XMET gene expression changes through different life stages compared to adult controls, the age at which most acute, subchronic and chronic studies begin chemical exposure.

## Materials and Methods

### Animals and study design

All animal studies were conducted in accordance with guidelines established by the United States Environmental Protection Agency (US EPA) Office of Research and Development (ORD)/National Health and Environmental Effects Research Laboratory (NHEERL) Institutional Animal Care and Use Committee (IACUC) and approved by the IACUC (IRP-NHEERL/ECD/CTB/CJC(JSL)/2006-01-r3). Procedures and facilities were consistent with the recommendations of the 1996 National Research Council (NRC) “Guide for the Care and Use of Laboratory Animals”, the Animal Welfare Act, and Public Health Service Policy on the Humane Care and Use of Laboratory Animals.

Timed-pregnant C57BL/6J or C3H/HeJ dams (n = 6) or male C57BL/6J mice, at approximately 6 (n = 5), 12 (n = 6), 18 (n = 7) and 24 (n = 10) months of age were purchased from Charles River Laboratory (Raleigh, NC) and acclimated for 1 week. Replicates were individual mice. Mice were housed (1 per cage) in polycarbonate cages on Alpha Dry bedding with a 12 hour light/dark cycle. Room temperature was 70±2°F with a relative humidity of 50%. The basal diet was Ralston Purina 5001 (Ralston Purina Co., St. Louis, MO) and water was provided ad libitum. Pregnant dams were sacrificed at gestation day (GD) 19 and male pups (n = 7) were sacrificed by decapitation. Male mice from additional litters at ages post-natal day (PND) 7 (n = 4), PND32 (n = 6), and PND67 (n = 7) or approximately 6, 12, 18 and 24 months of age were sacrificed using CO_2_ asphyxiation. All necropsies started in the morning and were completed by the afternoon. Studies with the C3H/HeJ mice have been described previously [11]. Individual animals in the litter were regarded as units. Livers were removed, weighed, cubed and stored at −80°C until RNA isolation. All aspects of these studies were conducted in compliance with the guidelines of the Association for Assessment and Accreditation of Laboratory Animal Care (AAALAC) International and were approved by the US EPA/NHEERL IACUC.

### RNA Isolation

Total RNA was isolated from mouse livers according to the TriReagent procedure (Molecular Research Center, Cincinnati, OH) and cleaned using the Qiagen RNeasy mini RNA cleanup protocol (Qiagen, Valencia, CA). The integrity of each RNA sample was determined using an Agilent 2100 Bioanalyzer (Agilent, Foster City, CA), and RNA quantity was determined using a Nanodrop® ND-100.

### Microarray hybridizations

Liver gene expression analysis was performed according to the Affymetrix recommended protocol using Affymetrix Mouse Genome 430 2.0 GeneChips® containing probes for over 30,000 genes. Total RNA (5 µg per sample) was labeled using the Affymetrix® One-Cycle cDNA Synthesis protocol and hybridized to arrays as described by the manufacturer (Affymetrix®, Santa Clara, CA). The cRNA hybridization cocktail was incubated overnight at 45°C while rotating in a hybridization oven. After 16 hours of hybridization, the cocktail was removed and the arrays were washed and stained in an Affymetrix GeneChip® fluidics station 450 according to the Affymetrix-recommended protocol. Arrays were scanned on an Affymetrix GeneChip® scanner. Four mice per age group were examined and cRNAs from individual mouse livers were hybridized to individual chips.

### Analyses of Microarray data

Analysis of microarray data was performed using Rosetta Resolver® (Seattle, WA). Probe set expression levels were normalized using the Rosetta gene-specific error model. Differentially expressed genes (DEG) were identified using an error-weighted one-way ANOVA with a Benjamini-Hochberg false discovery rate (FDR) of 0.05. Principal components analysis (PCA) was performed using Rosetta Resolver®. Hierarchical clustering was performed using CLUSTER and visualized with TREEVIEW [12]. Genes which exhibited gender-dependent expression were identified from the .cel files from two published studies [13] [14] using identical procedures as described above. Biological analyses were done using the C57BL/6J strain. All data is MIAME compliant and the raw data, as well as a detailed description of the microarray experiment, is available through Gene Expression Omnibus at the National Center for Biotechnology Information at http://www.ncbi.nlm.nih.gov/geo/, as accession number GSE21716.

### Evaluation of Selected Genes by Real-Time RT-PCR

The levels of expression of selected genes were quantified using real-time reverse transcription–PCR (RT-PCR) analysis. Briefly, total RNA was reverse transcribed with murine leukemia virus reverse transcriptase and oligo(dT) primers. The forward and reverse primers for selected genes (available upon request) were designed using Primer Express software, version 2.0 (Applied Biosystems, Foster City, CA). The SYBR green DNA PCR kit (Applied Biosystems, Foster City, CA) was used for real-time PCR analysis. The relative differences in expression between groups were expressed using cycle threshold (Ct) values as follows. The Ct values of the genes were first normalized with β–actin and glyceraldehyde 3-phosphate dehydrogenase (GAPDH) of the same sample. Assuming that the Ct value is reflective of the initial starting copy and there is 100% efficiency, a difference of one cycle is equivalent to a two-fold difference in starting copy. Means and SE (*n* = 4) for RT-PCR data were calculated by Student's *t* test. The level of significance was set at *p*≤0.05.

## Results and Discussion

### Ontogeny of hepatic XMET gene expression changes through the life stages of the mouse

**Figure 1:**
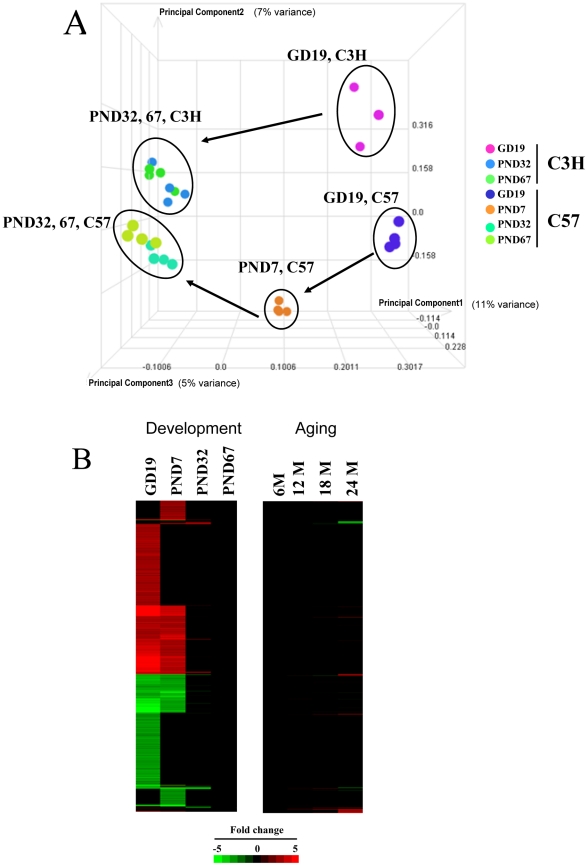
Transcriptional ontogeny of hepatic gene expression through life stages in the mouse. A. Principal components analysis (PCA) of the livers from mice at different life stages. The PCA shows the dramatic differences between the fetal/neonatal samples and the adult. More subtle differences were observed between adult and old mice (Not shown). C57, C57BL/6J; C3H, C3H/HeJ. B. Altered gene expression through life stages in the mouse liver. Differentially expressed genes (fold change≥|±2|) were identified and clustered as detailed in the Materials and Methods. There were no changes in gene expression in the “Adult” (PND67 and 6M) by definition. The intensity scale indicates fold-changes relative to the adult controls. Red, up-regulation; green, down-regulation; black, no change.

Hepatic gene expression profiles were generated using full genome gene arrays (Affymetrix MOE430_2 chips) from fetal (GD 19), neonatal (PND 7), prepubescent (PND32), middle age (12 months), and old (18 and 24 months) male C57BL/6J mice and GD19 and PND32 male C3H/HeJ mice. Gene expression was compared to 2 month old (PND67) animals for the fetal/neonatal experiments or 6 month old animals for the aged mice, as these studies were carried out at separate times. An unsupervised comparison by principal components analysis (PCA) between the liver profiles used in this analysis revealed the dramatic gene expression differences between the fetal and neonatal livers and their controls in the C57BL/6J mice (Figure 1A). Age segregates along principal component #1 axis (11% variance) and strain segregates along principal component #3 axis (5% variance). There were similar differences between the GD19 livers compared to the PND32 and PND67 livers from the C3H/HeJ mice. The differences between the 6, 12, 18 and 24 month samples were more subtle, but there were clear differences between 6 and 24 month old animals (not shown). The PCA clearly separated the samples from the two strains, not surprising given the known genetic and phenotypic differences between these strains, especially the known responses of the liver to hepatotoxicants [15]. The comparison also revealed differences between the mouse livers at PND67 and 6 months (data not shown) which may be due to a number of biological (e.g., age) or technical differences (e.g., sampling times). While there is evidence of diurnal effects on XMET gene expression [16], [17], this should not affect our results since all samples were collected during the day. The expression of all genes significantly altered (20,687 genes) is shown in Figure 1B, highlighting the dramatic differences in gene expression at GD19 and PND7. Interestingly, a smaller set of genes was differentially expressed only at PND7. In comparison, relatively few changes were observed in the aged mice.

**Figure 2:**
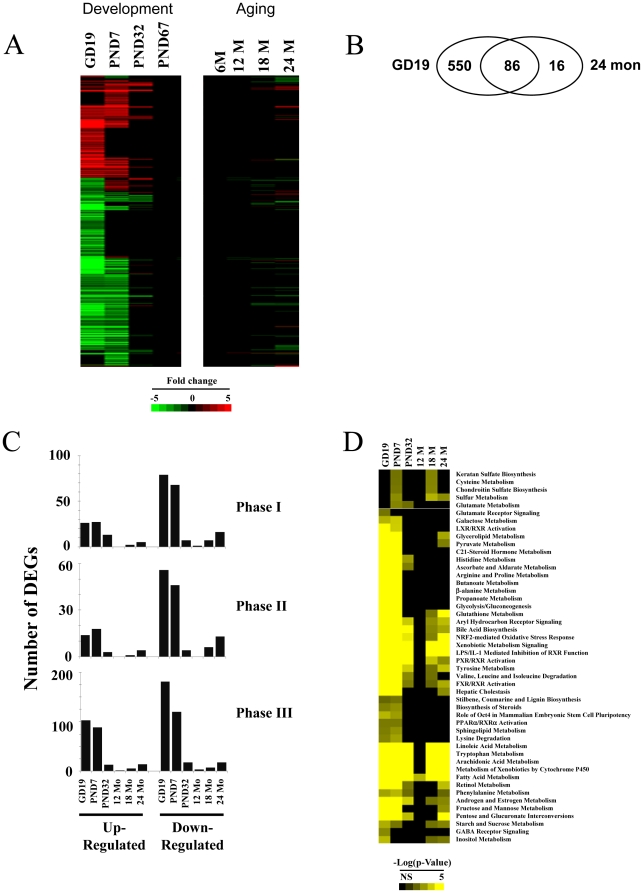
Altered expression of XMETs in the male mouse liver at different life stages. A. XMET expression at different life stages. XMET genes which exhibited significant differences in expression compared to adult animals were identified as detailed in the Materials and Methods. Genes were clustered using one-dimensional hierarchical clustering. B. Overlap in the XMET probe sets altered in the fetus (GD19) and at 24 months. C. Quantitation of the number of up- and down-regulated phase I, II and III probe sets at different life stages. DEGs, differentially expressed genes. D. Canonical pathways overrepresented by the XMET probe sets at different life stages. XMET genes described in Figure 2A were analyzed by Ingenuity Pathways Analysis. Pathways were clustered by one-dimensional clustering. Scales at the bottom indicate the −log(p-value) for all genes.

Expression of XMETs through life stages in the mouse was examined. XMETs were identified from a list provided in an earlier paper [18], as well as identified in the latest curation of the Affymetrix MOE430_2 chip. The list includes 190 phase I genes, 135 phase II genes, and 745 phase III genes. The list also includes 104 known regulators of XMET expression including nuclear receptors with known or putative roles in XMET regulation. Most of the XMETs (721 genes or ∼61% of total) were altered in at least one of the life stages (Figure 2A). Most of the changes occurred only in the fetus and neonate. More subtle changes were observed during aging. Most of the XMET genes altered at 24 months overlapped with those altered at GD19 (Figure 2B). Five probe sets including 4 genes (*Arntl*, *Slc6a15*, *Slc7a2*, *Gstt2*) were altered only at 24 months. The total number of XMET probe sets altered was 636, 500, 84, 5, 43, and 102 for GD19, PND7, PND32, 12 months, 18 months and 24 months, respectively. At GD19 and PND7, down-regulated genes outnumbered up-regulated phase I-III genes (Figure 2C). At PND32 up-regulated phase I genes outnumbered those that were down-regulated, whereas there were about equal numbers of up- and down-regulated phase II and III genes at this age. There were more down-regulated than up-regulated phase I and II genes at 24 months. A list of the expression changes of the XMETs is included in Table S1.

Using Ingenuity Pathways Analysis (IPA), canonical pathways that were significantly altered (p-value≤0.01) at the different life stages were identified. All differentially expressed XMETs were used as input for each life stage (Figure 2D). Most of the pathways (29) were significantly altered in fetal/neonatal and aged groups. Nineteen pathways were unique to the fetal/neonatal groups. No pathways were unique to the aged groups. This analysis demonstrates that the fetal and neonatal life stages exhibit profound differences in their XMET expression compared to adult mice, while the livers from aged mice exhibit more subtle differences in XMET expression.

### Gender-dependent changes in XMET expression at different life stages

**Figure 3:**
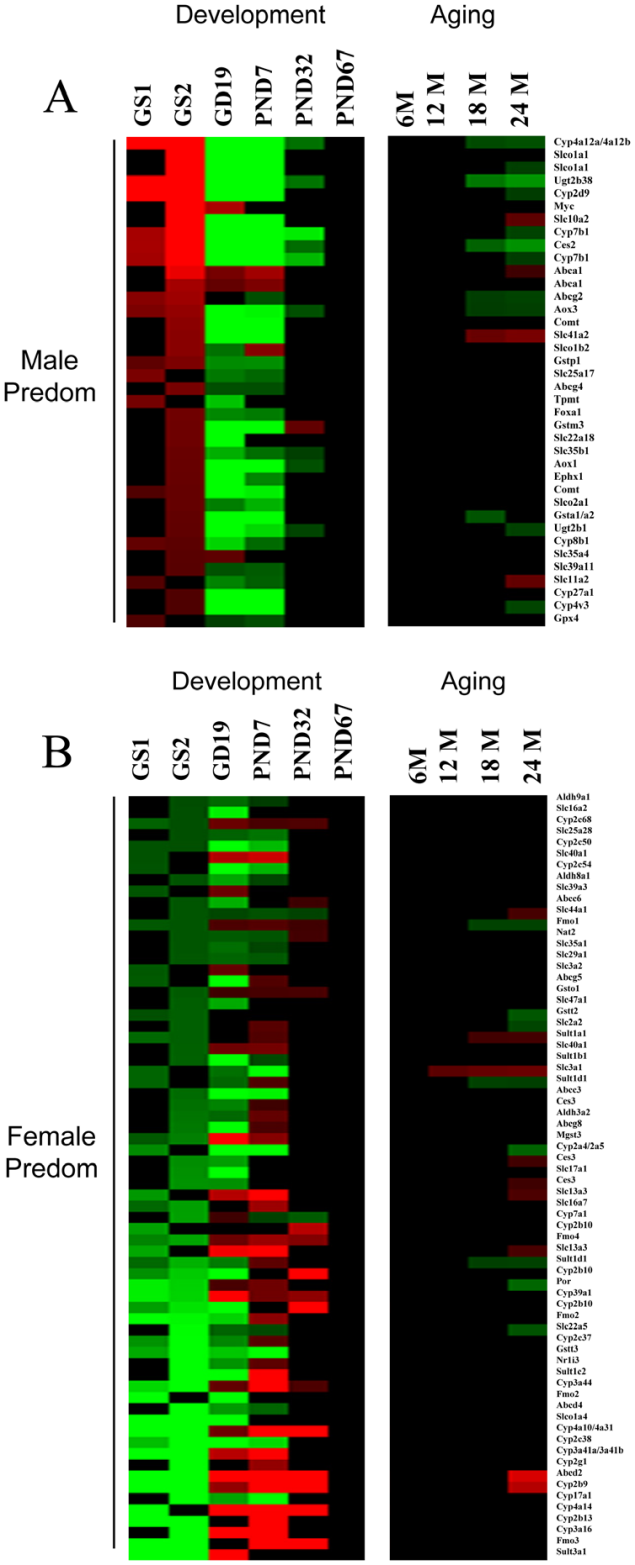
Life stage changes in XMET expression are partially gender-dependent. XMETs which exhibited gender differences in gene expression in the mouse liver in a reanalysis of two studies (gender study 1 (GS1), [14]; GS2, [13]) were examined for expression changes across life stage. Gender-dependent gene expression was calculated as a ratio of expression in males to that in females. At each life stage the expression in males and females was compared to adult baseline (PND67 and 6M). Probe sets predominantly expressed in males are indicated in red and female-predominant probe sets are indicated in green; intensity indicates the ratio of the gender difference. The comparison shows the suppressed expression of many male-predominant probe sets and increased expression of female-predominant probe sets in the fetus and neonate compared to the adults. A. Expression of male-predominant probe sets in the fetal and neonatal liver. B. Expression of female-predominant probe sets in the fetal and neonatal liver.

Gender-dependent gene expression in the rodent liver is determined in large part by the growth-hormone secretory pattern, which is continuous in females and pulsatile in males. Some XMET genes can exhibit dramatic gender differences in expression, and these gender-dependent patterns determine responses to pharmaceutical agents as well as environmentally-relevant chemicals [19]. The behavior of gender-dependent XMET genes through mouse life stages has not been previously assessed. We examined the expression of 106 XMET probe sets (out of the 721 XMET probe sets exhibiting altered expression at one or more time points) that also exhibited gender-dependent gene expression in the mouse liver. Gender-dependent genes were identified from two studies in which hepatic gene expression was compared between male and female adult mice [13] [14]. Gender-dependent gene expression was calculated as a ratio of expression in males to that in females. In male mice, most of the male-predominant genes were suppressed in the fetus and neonate and did not achieve full adult levels until after PND7 (Figure 3A). The genes included the male-specific testosterone 16alpha-hydroxylase, *Cyp2d9*. A number of the XMETs in male mice exhibited a fetal/neonatal-like expression as late as PND32 including *Aox1*, *Aox3*, *Ces2*, *Cyp4a12a/Cyp4a12b*, *Cyp7b1*, *Slc35b1*, *Ugt2b1*, and *Ugt2b38* (Figure 3A). Only four of the male-specific genes in male mice exhibited increased expression during development relative to adults including a transcription factor involved in cell proliferation (*Myc*) and three phase III genes (*Abca1*, *Slco1b2* and *Slc35a4*).

Many female-predominant genes exhibited a unique pattern of expression during development that included increased expression between GD19 and PND32, generally peaking at PND7 compared to the adults (Figure 3B). These included many classical female-specific genes involved in xenobiotic metabolism (e.g., *Abcd2, Cyp3a41a/Cyp3a41b, Cyp4a10/Cyp4a31, Fmo2, Fmo3*) as well as the testosterone hydroxylases *Cyp2b9* and *Cyp2b10* and the nuclear receptor *Nr1i3 (CAR)*. Many of the female-predominant genes (e.g., *Cyp17a1, Cyp2a4/Cyp2a5, Cyp2c38*) did not follow the pattern of higher fetal/neonatal expression compared to adults. These genes exhibited decreased expression between GD19 and PND32 relative to adults indicating they are under different control mechanisms during development.

Aging had more subtle effects on gender-dependent XMETs. At 24 months, 11 out of the 15 male-predominant probe sets that were altered in male mice exhibited decreased expression relative to 6 month old adults (*Abcg2, Aox3, Ces2, Cyp2d9, Cyp4a12a/Cyp4a12b, Cyp4v3, Cyp7b1, Slco1a1, Ugt2b1*, and *Ugt2b38*) (Figure 3A). Four male-predominant phase III probe sets (*Abca1, Slc10a2, Slc11a2, Slc41a2*) exhibited increased expression at 24 months. Nine out of 17 female-predominant probe sets that were altered exhibited increased expression at 24 months including *Abcd2, Ces3, Cyp2b9, Slc13a3, Slc3a1, Slc44a1*, and *Sult1a1* while the rest (*Cyp2a4/Cyp2a5, Fmo1, Gstt2, Por, Slc22a5, Slc2a2, Sult1d1*) were down-regulated. The pattern of general decreases in male-predominant genes indicates a general feminization of the liver XMET transcriptome during aging. These results are consistent with the feminization of gene expression observed in the aging rat liver [10], which likely originates from decreases in circulating testosterone and disruption of the growth hormone pulsatile secretion pattern in males [20].

STAT5b is an important determinant of growth hormone-mediated male-predominant gene expression [19], whereas STAT5a expression determines in part female-predominant gene expression [21]. Gender-predominant genes have been classified based on their expression behavior in wild-type vs. STAT5a-null or STAT5b-null mouse livers [19] [21]. As these studies examined gene expression in young adult mice (6–9 weeks), we compared the gender-predominant XMET genes identified in our studies to determine if STAT5a or STAT5b may be playing a role in the development of the male-specific profile. Out of the 106 XMETs identified that were expressed in a gender-predominant manner, 51 and 55 probe sets overlapped with the genes cataloged in the STAT5b-null or STAT5a-null studies, respectively. Almost all of the male-predominant genes from our analysis were classified as male-specific and were up-regulated by STAT5b in males [19], i.e., expression was negatively affected in STAT5b-null mice compared to wild-type mice. The genes included *Ces2, Gstp1, Foxa1, Cyp2d9, Slco1a1, Abcg2*, and *Cyp7b1*. Most of the male-predominant genes were unaffected in STAT5a-null mice [20]. Likewise, the female-predominant genes that were transiently up-regulated during development were classified as female-specific and down-regulated by STAT5b in male mice (*Abcd2, Aldh3a2, Ces3, Cyp2b10, Cyp2b9, Cyp2c37, Cyp39a1, Cyp3a16, Cyp4a10, Fmo1, Fmo2, Mgst3, Nr1i3, Por, Slc16a7, Sult1a1, Sult3a1*). Most of the genes in this category were unaffected by loss of STAT5a. Thus, this comparison provides evidence that STAT5b but not STAT5a is playing a role in the development of the male-specific signature between PND7 and PND67.

These results indicate that the immature male mouse liver exhibits characteristics of a feminized mouse including suppression of male-predominant genes. Many of these genes retain fetal/neonatal expression patterns until PND32, about the time of increases in circulating testosterone levels and sexual maturity. This is in contrast to the majority of fetal/neonatal genes which obtain adult levels by PND7. We uncovered a group of unique female-predominant XMETs in male mice that were transiently up-regulated relative to adults with peak expression at ∼PND7. Our comparison with the extensive microarray analysis of expression changes in STAT5a- and STAT5b-null mice indicated that STAT5b but not STAT5a plays a major role in driving the male-predominant expression pattern during development of the neonatal liver. It was curious that a smaller group of female-predominant genes exhibited suppressed expression throughout development (e.g., *Abcc3, Abcd4, Ces3, Cyp17a1, Cyp2a4, Cyp2c38, Cyp2c50, Cyp2c54, Fmo2, Gstt2, Gstt3, Slc17a1, Slc22a5, Slc29a1, Slco1a4, Sult1b1*) and thus were likely not under control of STAT5b. These genes, like many genes suppressed during development (Lee et al., submitted) may require factors other than STAT5b to achieve adult-like expression. Based on these results we would predict that immature male mice ∼PND7 would exhibit responses to drugs and xenobiotics similar to female mice.

### Impact of life stage on XMET gene family expression

The impact of life stage on the expression of families of XMETs was examined. Members of the alcohol dehydrogenase (*Adh*) enzyme family metabolize a wide variety of substrates, including ethanol, retinol, other aliphatic alcohols, hydroxysteroids, and lipid peroxidation products. Aldehyde dehydrogenase (*Aldh*) is the second enzyme of the major oxidative pathway of alcohol metabolism. Most of the *Adh* and *Aldh* family members exhibited suppressed expression relative to adults (Table S2). However, *Adh7, Adhfe1, Aldh1a3, Aldh1b1, Aldh1l2, Aldh3a2, Aldh3b1, Aldh4a1* and the 5 probe sets of *Aldh18a1* (encoding a glutamate gamma-semialdehyde synthetase involved in arginine and proline metabolism) were up-regulated during at least one age before maturity. At 24 months, *Adh4* and *Aldh3b1* were slightly elevated.

A large number of *Cyp* genes were altered during development (Table S3). While most *Cyps* were suppressed during development, a number of subclasses exhibited increased expression including members of the *Cyp2b* (i.e., *Cyp2b9, Cyp2b10, Cyp2b13*), *Cyp3a* (i.e., *Cyp3a16, Cyp3a41a/3a41b, Cyp3a44*), *Cyp4a* (i.e., *Cyp4a14, Cyp4a29, Cyp4a31*) and *Cyp4f* (i.e., *Cyp4f16, Cyp4f16/4f37, Cyp4f18*) subclasses. *Cyp* genes involved in bile acid biosynthesis were altered during development including the up-regulation of *Cyp39a1* and *Cyp7a1* and the down-regulation of *Cyp7b1* and *Cyp8b1*. A smaller subset of *Cyp* genes was altered by aging but in general, they exhibited smaller fold-changes than the more dramatic changes observed during development. Like the developmental stages, most of the *Cyps* altered with aging exhibited down-regulation except *Cyp2b9, Cyp3a13*, and *Cyp4f16*.

We also observed the suppression of carboxylesterase (*Ces*) genes during development. These included *Ces1*, *Ces2*, *Ces3*, *Ces5*, *Ces6*, and *Ces7*. *Ces3* exhibited a slight increase in expression at PND7 and 24 months. Carboxylesterases are important in the detoxification of organophosphorous pesticides [22] and pyrethroid insecticides [23], leading to the prediction of increased levels during development upon exposure.

The aldo/keto reductase (*Akr*) superfamily involved in phase I metabolism consists of more than 40 known enzymes and proteins. These enzymes catalyze the conversion of aldehydes and ketones to their corresponding alcohols by utilizing NADH and/or NADPH as cofactors. The enzymes display overlapping but distinct substrate specificity [24]. *Akr* genes that were up-regulated at GD19 and/or PND7 included *Akr1b3, Akr1b7, Akr1b8, Akr1c18* and *Akr1c20* (Table S4). *Akr1b3* and *Akr1b7* encode prostaglandin F2α synthase [25] and *Akr1c18* encodes a 21-hydroxysteroid dehydrogenase [26]. *Akr1c12* and *Akr1c13* were down-regulated at 24 months. *Akr1c12/13* functions as a dehydrogenase for endogenous hydroxysteroids [27].

Cytosolic and membrane-bound forms of glutathione S-transferase (*Gst*) are encoded by two distinct supergene families. These enzymes function in the detoxification of electrophilic compounds, including carcinogens, therapeutic drugs, environmental toxicants and products of oxidative stress, by conjugation with glutathione. Most of the *Gst* genes were down-regulated during development (Table S4). Increased susceptibility to liver carcinogenesis in mice and humans has been linked to single nucleotide polymorphisms in the *Gstt1* or *Gsta4* genes [28] that were down-regulated in our study. *Gstcd, Gstm5, Gsto1, Mgst2* and *Mgst3* were up-regulated. *Mgst2* and *Mgst3* catalyze the committed step in the biosynthesis of cysteinyl-leukotrienes (cys-LTs), potent smooth muscle contracting agents which play key roles in inflammatory and allergic diseases [29]. *Gpx3, Gpx7*, and *Gss* involved in glutathione synthesis were also up-regulated during development. At 24 months, there was weak up- (*Gsta3, Gstm3*) and down- (*Gpx7, Gstt2*) regulation of some family members.

Sulfotransferase (*Sult*) enzymes catalyze the sulfate conjugation of many hormones, neurotransmitters, drugs, and xenobiotic compounds. *Sult1a1, Sult1c2, Sult1d1* and *Sult3a1* were up-regulated and *Sult1b1* and *Sult5a1* were down-regulated during development (Table S4). *Sult1a1* was up-regulated and *Sult1d1* and *Sult5a1* were down-regulated at 24 months.

UDP-glucuronosyltransferases (UGT) transform small lipophilic molecules such as steroids, bilirubin, hormones, and drugs, into water-soluble, excretable metabolites. The UGT1A enzymes are encoded at a single locus that includes thirteen unique alternate first exons followed by four common exons. The UGT family members altered during development were universally down-regulated (Table S4). A smaller number of *Ugts* were altered at 24 months including up-regulation of *Ugt3a1* and down-regulation of *Ugt2a3, Ugt2b1, Ugt2b5, Ugt2b37* and *Ugt2b38*.

Expression of transporters located on both basolateral and apical membranes of hepatocytes was examined. There were more genes involved in transport that were under-expressed than over-expressed during development (Table S5). Transporters with increased fetal expression included genes with known endogenous functions such as transport of amino acids (*Slc1a5, Slc38a1, Slc38a5, Slc3a2, Slc43a1, Slc7a1*), adenine nucleotide (*Slc25a4*), glucose (*Slc2a1, Slc2a3*), heme (*Abcb10, Slc25a37, Slc25a38*, all found on the inner mitochondrial membrane), inorganic anion (*Slc4a1(erythrocyte membrane protein band 3, Diego blood group)*), inorganic phosphate (*Slc20a1*), monocarboxylic acids such as lactate (*Slc16a1*), urea (*Slc14a1*), and zinc (*Slc39a5, Slc39a8*). Many of these genes may be expressed in resident hematopoietic cells including nucleated erythrocytes (Lee et al., submitted).

Some of the phase III genes that are coordinately up-regulated during development may play essential roles in liver growth. For example, the amino acid transporters *Slc1a5*, *Slc7a5*, and *Slc3a2* play roles in regulating the target of rapamycin complex 1 (*Torc1*), a highly conserved serine/ threonine kinase that in mammals activates cell growth in response to stimuli including nutrients (amino acids), growth factors (such as insulin and insulin-like growth factor), and cellular energy status (ATP). Inhibition of TORC1 activates autophagy [30]. L-glutamine uptake is regulated by *Slc1a5* and loss of *Slc1a5* function inhibits cell growth and activates autophagy. The complex of *Slc7a5*/*Slc3a2*, acts as a bidirectional transporter that regulates the simultaneous efflux of L-glutamine out of cells and transport of L-leucine/essential amino acids into cells. Thus, the increases in *Slc1a5* and *Slc3a2* in the fetus may be linked to coordinated cell growth and proliferation through mTOR [31].

A number of transporters exhibited increased abundance in aged mice that may be associated with tertiary lymphoid neogenesis (TLN), a phenomenon entailing formation of ectopic lymphoid structures observed in chronically inflamed tissues [32]. In the aging mouse liver, macrophages, T cells, B cells and neutrophils form foci in the periportal region of the liver lobule [33]. Two of the transporters (*Abca7, Abcg3*) are either lymphoid-specific or associated with inflammation [34] [35]. Other transporters with increased abundance included *Abcd2* involved in the peroxisomal import of fatty acids and fatty acyl-CoAs, *Slc41a2*, a magnesium transporter and two amino acid transporters (*Slc3a1, Slc7a1*).

The expression of known and putative transcriptional regulators of the XMETs was also examined. The nuclear receptor superfamily controls basal and chemical-inducible regulation of a number of XMETs [36]. While most of the nuclear receptors were down-regulated during development, *Nr1i3* (CAR), *Nr2c2* (known as TR4 or TAK1), *Nr2f1* (COUP-TF1), and *Nr3c1* (glucocorticoid receptor, GR) were up-regulated at one or more time points during development (Table S6). Aging resulted in up-regulation of *Nr1h2* (liver X receptor beta, LXRbeta) and down-regulation of *Nr1d2* (Rev-erb beta).

Transcriptional regulators that fall into other transcription factor families were examined. The heterodimeric partners encoded by the *Ahr* and *Arnt* genes which control the transcriptional response to polyaromatic hydrocarbons like dioxin were up-regulated during development (Table S6). Activation of the Kelch-like ECH-associated protein 1 (KEAP1)-NF-E2-related factor 2 (NRF2)-signaling pathway is an adaptive response to environmental and endogenous stressors and serves to render animals resistant to chemical carcinogenesis and other forms of toxicity, while disruption of the pathway exacerbates these outcomes [37]. We observed increased expression of *Nrf2* (*Nfe2l2*) and down-regulation of *Keap1* (−1.8 fold-change at GD19) during development. Heterodimeric partners of Nrf2 were up- (*Maff* and *Mafk*) or down- (*Mafg*) regulated. Other *Maf* and *Nrf* family members were also up-regulated during development. *Arnt1*, *Maf* and *Nfe2* were up-regulated and *Mafb* and *Maff* were down-regulated at 24 months. The fetal and neonatal life stages may exhibit induced *Nrf2* and decreased *Keap1* expression to help protect the fetus or newborn from environmental stressors including oxidative stress.

The expression of ∼40 genes at all life stages was examined by RT-PCR. Two genes (α-fetoprotein (*Afp*) and *Cd34*) exhibited strong expression at GD19 and PND7 compared to adults (data not shown), consistent with their known fetal expression in the liver. The RT-PCR results for the XMETs were consistent with those observed by microarrays (Table S7). Additional genes not examined by microarray were queried by RT-PCR including sulfotransferase family 2A, dehydroepiandrosterone (DHEA)-preferring, member 1 (*Msta1*) which was dramatically up-regulated only at PND7. Two 17β-hydroxysteroid dehydrogenases (*Hsd17b5* and *Hsd17b7*) were down-regulated at GD19 and PND7. In an examination of transcription factors important in XMET expression, *Ahr*, *Car* and *Pxr* genes exhibited decreased expression at one or more time points during development while *Ppara* was up-regulated at PND7. The expression of *Ppara* correlates with the up-regulation of target genes *Cyp4a10* and *Cyp4a14* while the down-regulation of *Car* and *Pxr* correlates with the down-regulation of many XMETs that are controlled by these transcription factors. We also confirmed the up-regulation of *Cyp2b9* and down-regulation of *Cyp1a2* and *Cyp7b1* at 24 months (data not shown).

Our comprehensive analysis of XMET expression in mice revealed key features of XMET expression at different life stages. A large number of XMETs (∼61% of total) were altered in the fetus and/or neonate, and most of these genes including phase I–III genes were underexpressed relative to adults. Expression of many of XMETs achieved adult levels by PND7-PND32. Consistent with our findings, a number of published studies indicate that in mice and rats, specific XMETs are generally underexpressed during development and exhibit increased expression from before birth to adulthood. Choudhary et al. [38] identified *Cyp* genes that were increased during development when assessed in whole fetuses from mice. In rats, there was a 4- and 6- fold increase in CYP protein content at PND7 and PND14, respectively, compared with PND1 [39]. In rats, *CYP1A1* was expressed during early gestation, but expression of most of the other CYP enzymes occurred at or near birth (*CYP2B*, *CYP2C23*, *CYP3A*) or immediately after birth (*CYP2E1*) [40]. *CYP1A2*, *CYP2C6*, *CYP2C11*, *CYP2C12*, and *CYP4A10* were expressed only after the first week of birth [40] [41] [39] [42]. *CYP2B1* activity at PND4 was comparable to levels observed in adult livers [39], whereas postnatal [40] activity of *CYP2E1* increased linearly with age and at PND32 was comparable to that in adult liver [40] [39]. Based on the pattern of XMET expression, we would predict that the fetus and neonate would be more susceptible to chemicals that do not require metabolic activation (and thus *Cyp* expression) for toxic effects. Increased sensitivity in the fetus and neonate may be compounded by the fact that the dam also exhibits changes in XMET expression. The expression of a number of XMETs decreased in the livers of F344 rat dams during late-term pregnancy and lactation [43]. Thus, the fetus and neonate may be more sensitive to xenobiotic exposure not only because of XMET changes in the livers of the fetus or neonate but also because of decreases in the efficient inactivation and excretion of toxic or potentially toxic chemicals by the dam.

Analysis of XMET expression in the livers from old mice indicate differences in phase I–III expression consistent with that observed previously in rats [9] [10]. Compared to the fetus and neonate, the changes in the aged mice were more subtle. Although most of the phase I and III genes were down-regulated by aging, most of the phase II genes were up-regulated. Recently, expression of a large number of XMETs was examined in the livers from aged male C57BL/6J mice [42] and many of the changes that we observed in our microarray study were confirmed by RT-PCR in this study. These included down-regulation by aging of *Cyp1a2, Cyp4a12, Gsta1/a2, Gsta4, Gstt2, Sultd1, Ugt2a3, Ugt2b1* and up-regulation by aging of *Sult1a1*.

Regarding the regulation of environmentally-relevant chemicals, the *US EPA's Draft Guidelines for Carcinogen Risk Assessment*, requires that if data are available from an epidemiological study on the effects of childhood exposure or an animal bioassay involving early-life exposure, a risk estimate that includes childhood exposure should be developed [44] [45]. Cancer risks are considered higher from early-life exposure than from similar exposure durations later in life based on extensive literature in animals [45]. Risk estimates that pertain to childhood exposures are usually adjusted, i.e., for exposures before 2 years of age, a 10-fold adjustment factor is invoked, while for exposures between 2 and <16 years of age, a 3-fold adjustment factor is used. No adjustments are made for individuals 16 years and older including aging adults. These adjustment factors do not necessarily reflect the underlying basis for differences in xenobiotic metabolism and responses to carcinogens between life stages. Our analysis of gene expression in the livers of mice at different life stages is one step to determine if the adjustment factors are too simplistic, chemical class-specific or adequately protective of sensitive populations. Future work will be directed towards determining the chemicals to which different life stages may exhibit altered responses compared to adults. For the fetus, responses will depend in part on effects of tissues that act as a metabolic barrier to environmental exposure to protect the embryo (yolk sac) and the fetus (placenta). For the neonate, xenobiotic metabolism in the maternal liver as well as any in the mammary gland will need to be considered.

### Summary

In the presence of foreign compounds, metabolic homeostasis of the organism is maintained by the liver's ability to detoxify and eliminate these xenobiotics. This is accomplished, in part, by the expression of XMETs, which metabolize and transport xenobiotics and determine whether exposure will result in altered responses. This project was designed to examine the changes in XMET mRNAs from early to late life stages in male C57BL/6J mice. Differences with adults in XMET expression were striking in the fetus and neonate (GD19 and PND7). The livers from aged mice exhibited more subtle differences in their XMET expression compared to young adults. In general, the majority of XMETs altered during development were underexpressed. Our results also showed that the developing male mouse fetus exhibits characteristics of a feminized mouse including suppression of male-predominant probe sets. This comprehensive catalog of XMET hepatic gene changes through the life stages of the mouse is being used to predict differences in sensitivity to chemicals at different life stages (Lee et al., in preparation).

## Supporting Information

Table S1 **XMET genes altered with life stage.**
(XLSX)

Table S2 **Altered expression of alcohol dehydrogenase (** ***Adh*** **) and aldehyde dehydrogenase (** ***Aldh*** **) family members.**
(XLSX)

Table S3 **Altered expression of** ***Cyp*** **family genes.**
(XLSX)

Table S4 **Impact of life stage on phase II metabolism genes.**
(XLSX)

Table S5 **Impact of life stage on phase III transporter genes.**
(XLSX)

Table S6 **Impact of life stage on transcriptional regulators of XMET expression.**
(XLSX)

Table S7 **RT-PCR confirmation of microarray results.** Expression of xenobiotic metabolism genes from GD19-PND67 was determined by RT-PCR. Significant fold-changes (p≤0.05) are indicated.
(XLSX)
